# Local field potentials in major depressive and obsessive-compulsive disorder: a frequency-based review

**DOI:** 10.3389/fpsyt.2023.1080260

**Published:** 2023-04-26

**Authors:** Wei Zhang, Botao Xiong, Yang Wu, Linglong Xiao, Wei Wang

**Affiliations:** Department of Neurosurgery, West China Hospital, Sichuan University, Chengdu, Sichuan, China

**Keywords:** deep brain stimulation, obsessive–compulsive disorder, major depressive disorder, local field potentials, mechanisms, frequency bands

## Abstract

**Objectives:**

The purpose of this paper is to provide a mini-review covering the recent progress in human and animal studies on local field potentials (LFPs) of major depressive disorder (MDD) and obsessive-compulsive disorder (OCD).

**Materials and methods:**

PubMed and EMBASE were searched to identify related studies. Inclusion criteria were (1) reported the LFPs on OCD or MDD, (2) published in English, and (3) human or animal studies. Exclusion criteria were (1) review or meta-analysis or other literature types without original data and (2) conference abstract without full text. Descriptive synthesis of data was performed.

**Results:**

Eight studies on LFPs of OCD containing 22 patients and 32 rats were included: seven were observational studies with no controls, and one animal study included a randomized and controlled phase. Ten studies on LFPs of MDD containing 71 patients and 52 rats were included: seven were observational studies with no controls, one study with control, and two animal studies included a randomized and controlled phase.

**Conclusion:**

The available studies revealed that different frequency bands were associated with specific symptoms. Low frequency activity seemed to be closely related to OCD symptoms, whereas LFPs findings in patients with MDD were more complicated. However, limitations of recent studies restrict the drawing of definite conclusions. Combined with other measures such as Electroencephalogram, Electrocorticography, or Magnetoencephalography and long-term recordings in various physiological states (rest state, sleep state, task state) could help to improve the understanding of potential mechanisms.

## Introduction

Major depressive disorder (MDD) is a common psychiatric disorders (1), characterized by symptoms including depressed mood or anhedonia (2), affecting approximately 6% of the population worldwide (3). About 30% of this disease becomes treatment-resistant depression (TRD) among patients with major depression.

It is estimated that 2–3% of the population suffer from obsessive–compulsive disorder (OCD) (4), characterized by distressing intrusive thoughts (obsessions) and often time consuming repetitive behaviors (compulsions) (5). Apart from obsessions and compulsions, substantial comorbidity, anxiety disorders, mood disorders, impulse-control disorders, and substance use disorders are common in OCD, with patients feeling confused in relationships and social functioning domains (6). However, 10% of patients are not relieved with sufficient drugs and long duration (7).

In 1999, Deep brain stimulation (DBS) targeting the anterior limb of the internal capsule (ALIC) was found to be beneficial for intractable OCD (8), and many reports about other targets of DBS in patients with OCD have been published. Moreover, DBS had been proven to produce clinical benefits in patients with TRD since 2005 (9). With direct access to the subcortical activity, local field potentials (LFPs) has provided valuable insight into disease mechanisms and cognitive processes. It is known that aberrant activity in cortico-basal ganglia-thalamo-cortical loops may be involved in the symptoms, but the specific relationship between LFPs and OCD is unclear. Likewise, the relationship between LFPs and MDD is unknown.

This review aimed to summarize the changes of LFPs in patients with OCD and MDD. In addition, we expect to point out how electrophysiology research in patients with DBS will continuously offer new information with mechanism when combined with LFPs.

## Local field potentials

As an aspect of neural activity in the brain, LFPs are increasingly used as a reflection of the ongoing transmission through neural networks (10), which can be recorded using metal, glass electrodes, or silicon probes inserted in the deep brain areas during or after operation (11). In fact, LFPs have been neglected for a few decades because *in vivo* neurophysiological research focused mostly on isolating action potentials from individual neurons. They are of interest to researchers who study cortical function in recent years, because LFPs provide a unique window to integrative excitatory and inhibitory synaptic processes for neural population activity (12, 13).

According to the changes of frequency, LFP bands were defined as follows: delta (0–3 Hz), theta (4–7 Hz), alpha (8–12 Hz), beta (13–35 Hz), gamma (31–200 Hz), and high-frequency oscillation (>200 Hz) (14). Different frequency-band oscillations reflect diverse neural processing pathways and contributions of transmembrane current from cellular activities. Moreover, LFPs can provide stable signals for long-term chronic experiments or clinical applications.

Therefore, LFPs provide more chances to explore the electrophysiological mechanism of psychiatric disorders. It is known that the presence of abnormal beta bursts is significantly correlated with the degree of motor impairment and the severity of rigidity/bradykinesia in Parkinson’s disease (PD) (15). However, there are few reports on the electrophysiological characteristics of OCD and MDD.

## LFPs in MDD

### Theta frequency

In a study by Cervera-Ferri et al., stimulating electrodes were implanted in the subgenual cingulate gyrus (SCG) in urethane-anesthetized rats bilaterally, while recording electrodes were placed at the dorsal hippocampus and basolateral amygdala. After 1 h stimulation of SCG, the power of slow wave (SW, <1.5 Hz) and theta (3–12 Hz) frequencies in the hippocampus and basolateral amygdala increased with positive results in refractory depression (16). However, the oscillatory changes detected might not be equivalent to those observed in the awake rats, which cannot reflect the real changes after long stimulation periods as they are obtained shortly after DBS offset.

### Alpha frequency

Neumann et al. found that the increased alpha-activity in the bed nucleus of the stria terminalis (BNST) showed a positive correlation with the level of depression and aberrant alpha-band activity in MDD might be reduced by DBS (17).

### Beta frequency

Merkl et al. evaluated the LFP changes during multifaceted empathy test in patients who received DBS in the subcallosal cingulate cortex (SCC). The study revealed that beta desynchronization for empathic involvement was associated with self-reported severity of depression (18). Moreover, early antidepressant response during intraoperative stimulation was linked with a decrease in beta power in eight patients who received SCC-DBS (19). Clark et al. also reported that beta power in subgenual anterior cingulate cortex (sgACC) was inversely related to HAMD score in MDD patients (20).

### Gamma frequency

DBS in the left ALIC resulted in a broad power increase in the right BNST, including significant increases in low and high gamma power, and an overall improvement of symptoms (21). Zhang et al. reported seven patients underwent Habenula (HB)–DBS surgery and found the LFP asymmetry in the left and right HB. Specifically, the power of the high-beta oscillation (21–30 Hz) in the left HB presented the largest negative connection with the patients’ baseline HAMD scores. While the most distinct correlations occurs between the power of both the gamma oscillation (71–90 Hz) and the patients’ baseline HAMA scores in the right HB (22). Voget et al. compared the difference of LFP between the FSL rats and Flinders resistant line (FRL) rats under urethane anesthesia, and found decreased oscillatory activity in the low gamma band in vmPFC and nucleus accumbens(Nacc) of FSL rats, with increased activity in the subthalamic nucleus (STN) (23). In a rat model of depression, a unipolar stimulating electrode was inserted into right vmPFC, while recording electrodes were implanted bilaterally into vmPFC and hippocampus. After DBS treatment, LFP oscillations in beta and gamma bands increased in vmPFC and hippocampus, with increased coordinated activity between them (24). In 14 consecutive patients who underwent bilateral SCC-DBS lead implantation, Smart et al. found that asymmetric power spectral density changed after acute unilateral SCC stimulation. Left stimulation induced broadband ipsilateral decrease in theta, alpha, beta, and gamma bands and right stimulation effects were restricted to ipsilateral beta and gamma bands decrease (25). Low band frequencies, typically between 2 and 20 Hz, were observed in SCC and adjacent targets, evolving with stimulation (26).

## LFPs in OCD

### Theta frequency

Acute high frequency stimulation reduced theta band activities, accompanied by other frequency bands increasing in BNST/ALIC and frontal cortex in four patients with OCD (27). In Winter et al.’s study, OCD symptoms subsided in one patient who received metacognitive therapy (MCT), with a decrease in theta band activity and an evident increase in alpha, beta, and gamma band activity in BNST/IC (28). Xiong et al. also reported a positive and consistent trend between clinical outcomes and theta-beta oscillation (29). Rappel et al. compared the LFPs between OCD and PD patients targeted in the subthalamic nucleus (STN), and they found that the ventral area of the STN displays distinct theta (6.5–8 Hz) oscillatory activity only in patients with OCD (30). Buot et al. recorded STN LFP activity while patients with OCD performed emotional categorization tasks, and discovered that modulations of STN theta band activity which related to emotional regulation were correlated with OCD symptoms severity (31).

### Delta frequency

Delta band power showed a strong negative correlation with OCD symptom intensity in the bilateral ventral capsule/ventral striatum (VC/VS), when intracranial electrophysiological data of VC/VS and BNST were recorded in three patients with OCD both in the clinic and natural environments (32). Bastin et al. reported that acute OCD symptoms might be related to abnormally high oscillatory activity in STN, particularly in the left hemisphere and delta-alpha (1–12 Hz) frequency (33).

### Other frequency

Wu et al. implanted electrodes bilaterally in the BNST and random control brain regions in 32 male Wistar rats and recorded corresponding LFPs during compulsive and noncompulsive behavior (34). During the initial phase of compulsion, delta oscillations increased, peaked during compulsion, and dropped afterwards. In contrast, gamma oscillations decreased before and during compulsion, and increased after compulsion. Beta oscillations increased when compulsion symptoms stopped. Furthermore, the percentage change of these bands during compulsion was strongly linked with the compulsive suppression effect of BNST electrical stimulation. Overall, few articles on LFP alterations in animals with OCD exist.

## Discussion

Different neuronal oscillations have always been considered as brain organizers, coordinating brain areas into networks in the normal or disordered state (35–38). To our knowledge, this was the first review to investigate LFP oscillations in patients with OCD and MDD. In this review, low frequency bands, especially the theta band, appeared more positively involved in modulating psychiatric-condition-related networks in patients with OCD (Table 1). However, such involvement may also be observed in levodopa-induced dyskinesias, dystonia, Tourette’s syndrome, schizophrenia, and attention deficit hyperactivity disorder (27). Meanwhile, changes in other frequency bands have also been reported. Since frequency bands are interrelated and interact with each other in psychiatric-disorder-related networks, changes in one frequency band activity may also influence the activity of other frequency bands. Here, critical caution is needed when considering the theta band as a biomarker in patients with OCD.

**Table 1:** Summary of recent studies on LFPs of OCD.

Author year type	Country participants (sample size)	Target laterality	Stimulation parameters	Primary measure	Main findings
Bastin et al. (33) (2014) Case report	France Patients (*N* = 2)	STN Bilateral	1.8 V 130 Hz 60 μs	Y-BOCS CGI GAF	Atypically high oscillatory activity in the STN, particularly delta-alpha (1–12 Hz) frequency range in the left hemisphere, may be linked to acute OCD symptoms.
Wu et al. (34) (2016) Animal trials	China Male Wistar rats (*N* = 32)	BNST Bilateral	0.5 mA 100 Hz 50 μs	Rat location in cage Drinking behavior	Delta oscillations showed a positive correlation with symptoms, while Gamma oscillations showed an opposite trend.
Rappel et al. (30) (2018) Case report	Israel Patients (*N* = 2)	STN Bilateral	0.5 V/1 V 120 Hz/130 Hz 60 μs	Y- BOCS	Theta oscillatory activity in ventral part of STN reduced when OCD symptoms were developed and negatively associated with the intensity of symptoms over time.
Winter et al. (28) (2019) Case report	Israel Patients (*N* = 1)	BNST/IC Bilateral	NA	Y- BOCS	MCT reduced the symptoms of OCD with a decrease in theta band activity and an increase in alpha, beta, and gamma band activity. MCT reduced theta frequency band synchronization between BNST/IC LFP and frontal brain EEG.
Buot et al. (31) (2020) Clinical trials	Israel Patients (*N* = 7)	STN Bilateral	NA	Y-BOCS	During image presentation, emotional modulation was mostly limited to low frequencies (2–8 Hz).
Provenza et al. (32) (2021) Case report	America Patients (*N* = 5)	VC/VS or BNST Bilateral	Varied	Y-BOCS Y-BOCS II	Delta-band power exhibited a strong negative correlation with OCD symptom intensity in bilateral VC/VS.
Schwabe et al. (27) (2021) Clinical trials	Germany Patients (*N* = 4)	BNST/ ALIC Bilateral	1 V/2 V/3.5 V 130 Hz 210 μs	Y-BOCS	Theta oscillatory decreased, while alpha, beta, and gamma bands activity increased in both the BNST/ALIC and the frontal EEG after acute high frequency stimulation of the BNST/ALIC.
Xiong et al. (29) (2021) Case report	China Patients (*N* = 1)	NAc/ALIC Bilateral	NA	HARS HDRS Y-BOCS	There was a positive and consistent relationship with compulsive symptoms and theta-beta oscillation.

ALIC, anterior limb of the internal capsule; BNST, bed nucleus of the stria terminalis; BNST/IC, bed nucleus of the stria terminalis/internal capsule; CGI, clinical global impression; GAF, global assessment of functioning; HARS, Hamilton anxiety rating scale; HDRS, Hamilton depression rating scale; LFP, local field potential; MCT, metacognitive therapy; NA, not available; NAc, nucleus accumbens; STN, subthalamic nucleus; Y-BOCS, Yale-brown obsessive compulsive scale.

In clinical studies, LFP findings in patients with MDD involved different frequency bands, unlike animal study observations. No clear conclusions could be drawn about the direction of the difference due to considerable inconsistencies between the study design and methodology. We concluded that the neuronal architecture of the mouse brain is different from that of the human. For example, the human hippocampus has a larger size and more neurons than rodents. Therefore, humans would have a longer period than animals to bring more cell assemblies to be linked synaptically in various brain processes such as memory and learning (39), showing lower frequency oscillations than rodents. Some researchers conjecture that the frequency of a neural oscillation has an inverse relationship with the size of a neuronal network (40–42).

The SCC LFP recordings in the bilateral hemispheres are asymmetric; left-sided stimulation resulted in widespread changes in the frequency bands, and the right-side stimulation effects were restricted to the beta and gamma bands (25). Similarly, the HB LFP recordings in the left hemisphere showed high-beta oscillation with depressive symptoms, while gamma oscillation was distinct in the right hemisphere (22). This lateralized alteration implied an asymmetric role of the bilateral hemispheres in modulating psychiatric-disorder-related networks, identical to the laterality of brain speech function.

However, current research has produced strikingly different results, as they were obtained through various targets and under different physiological states, which impeded arriving at definite conclusions. Furthermore, some studies did not use stimulation with the targets, and the stimulation parameters were not unified. As LFP is a partly ambiguous signal involving multiple neuronal processes, careful analytical modeling and empirical considerations are required to interpret the derived information.

Based on the findings, we conclude that synchronization in multiple bands, rather than just abnormalities in a single frequency band, may play an essential role in modulating OCD and MDD symptoms. In the future, we might consider modulating networks that support specific symptom patterns instead of focusing on a single, optimal gray matter target (43).

### Current challenges and future perspective

Despite great potential, there are still limitations in the current study. Firstly, recent studies have focused on different targets or nuclei, but they can produce different oscillation bands, which precluded a quantitative synthesis of the outcome. Apart from frequency, the LFP signals contain multiple dimensions, including waveform, power, phase, entropy, and coupling (14); developing advanced algorithms could help retrieve more pertinent information (44). Secondly, LFP oscillations were recorded in different timelines, such as intraoperatively and before or after DBS stimulation (19, 45); therefore, comparisons could not be performed, more studies with recording in the same timeline are needed in the future. Finally, because of the small sample sizes of patients (Tables 1, 2), more studies are needed to confirm the validity and reliability of LFPs. In addition, LFPs data are mostly recorded in the resting state, with few reports on the task (46) or sleep state (32), making it difficult to fully reflect the mechanisms of MDD and OCD.

**Table 2:** Summary of recent studies on LFPs of MDD.

Author year type	Country participants (sample size)	Target laterality	Stimulation parameters	Primary measure	Main findings
Neumann et al. (17) (2014) Clinical trials	Germany Patients (*N* = 14)	50% BNST 50% SCC Bilateral	NA	BDI HAMD	Higher alpha-power was found in MDD compared with OCD in the BNST area. The mean alpha-power was correlated with severity of depressive symptoms in MDD.
Voget et al. (23) (2015) Animal trials	Germany FSL and FRL rats (*N* = 12)	vmPFC Nacc shell STN Left	NA	NA	Oscillatory activity in the low gamma band decreased in the vmPFC and Nacc of FSL rats, while increased in STN.
Clark et al. (20) (2016) Clinical trials	Canada Patients (*N* = 14)	sgACC Bilateral	NA	HAMD HAMA	The sgACC beta power presented a negative correlation with depression severity in TRD patients.
Cervera-Ferri et al. (16) (2016) Animal trials	Spain Male Wistar rats (*N* = 6)	Infralimbic cortex Bilateral	130 Hz 60 μA 80 μs	NA	The power of slow wave (SW, <1.5 Hz) and theta (3–12 Hz) frequencies in the hippocampus and basolateral amygdala increased with positive results in refractory depression.
Merkl et al. (18) (2016) Clinical trials	Germany Patients (*N* = 9)	sgACC Bilateral	NA	BDI HAMD-24	Beta oscillations in the sgACC area were related to negative emotion sharing in TRD patients which can improved with chronic DBS.
Smart et al. (25) (2018) Clinical trials	America Patients (*N* = 18)	SCC Bilateral	130 Hz 90 μs 6–8 mA or 3.5–5 V	HDRS	Ipsilateral theta, alpha, beta, and gamma bands decreased after left stimulation, While right stimulation was limited to ipsilateral beta and gamma reductions.
Jia et al. (24) (2019) Animal trials	China Male SD rats (*N* = 34)	vmPFC and hippocampus Bilateral	LFS: 20 Hz 400 μA, 200 μs HFS: 130 Hz 100 μA, 90 μs	OFT FST SPT	Acute HFS and LFS DBS produced significant antidepressant-like effects, with beta and gamma bands increased in vmPFC and hippocampus, while coordinated activity also increased between them.
Frank et al. (21) (2021) Case report	America Patients (*N* = 1)	ALIC and BNST Bilateral	NA	VAS PHQ-9	Left ALIC stimulation resulted in a broad increase in power in the right BNST, with notable increases in low and high gamma bands, as well as a general improvement in symptoms.
Sendi et al. (19) (2021) Clinical trials	America Patients (*N* = 8)	SCC Bilateral	130 Hz 6 mA 90 μs	HDRS-17	Intraoperative exposure to therapeutic stimulation resulted in an acute decrease in symptoms of depression. There was a positive correlation between the decrease in intraoperative left beta power and HDRS.
Zhang et al. (22) (2022) Clinical trials	China Patients (*N* = 7)	Habenula Bilateral	1.6–3.45 V 60–160 Hz 60–120 μs	HAMD YMRS HAMA PSQI	The power of the high-beta oscillation (21–30 Hz) in the left HB and the patients’ baseline HAMD scores showed the strongest negative correlation. The power of the gamma oscillation (71–90 Hz) and the baseline HAMA scores had the distinct correlation in the right HB.

ALIC, anterior limb of the internal capsule; BNST, bed nucleus of the stria terminalis; BDI, beck depression inventory; FRL, flinders resistant line; FSL, flinders sensitive line; FST, forced swimming test; HAMA, Hamilton anxiety rating scale; HAMD, Hamilton Depression Scale; HAMD-24, HB, Habenula; HDRS, Hamilton depression rating scale; HFS, high frequency stimulation; LFS, low frequency stimulation; MDD, major depressive disorder; Nacc, nucleus accumbens; OCD, obsessive–compulsive disorder; OFT, open field test; PHQ-9, patient health questionnaire-9; PSQI, Pittsburgh sleep quality index; SCC, subcallosal cingulate cortex; SD, Sprague–Dawley; sgACC, subgenual anterior cingulate cortex; SPT, sucrose preference test; STN, subthalamic nucleus; vmPFC, ventromedial prefrontal cortex; VTA, ventral tegmental area; YMRS, youth mania rating scale.

As LFPs represent the multiple neuronal processes, how to filter and interpret the LFPs is a challenge for current researchers. Combined with other measures such as Electroencephalogram, Electrocorticography, or Magnetoencephalography, LFPs may provide more cortical information (27, 47–50). Future research using long-term LFP recordings in various physiological states (rest state, sleep state, task state) could help to improve the understanding of potential mechanisms.

## Conclusion

This mini-review was not comprehensive; however, it summarized the recent advances in LFPs of MDD and OCD. As mentioned above, LFPs provided more chances to understand the electrophysiological characteristics and explore the potential mechanisms. Low-frequency activity seemed closely related to OCD symptoms, whereas LFP findings in patients with MDD were complicated. While MDD in human had a close relationship with the alpha or beta frequency or gamma oscillations. All different frequency bands seemed to participate in MDD and OCD networks. In the future, other multiple measures with long-term LFP recordings in various physiological states (rest, sleep, and task states) could be applied in patients with OCD or MDD.

## Author contributions

WZ, BX, and WW proposed the conception and design of the study. WZ and BX conducted the literature search, study screening, data extraction, and quality assessment. WZ, BX, YW, LX, and WW were involved in the analysis and interpretation of data. WZ and BX drafted the manuscript and all authors revised it critically under the guidance of WW. All authors contributed to the article and approved the submitted version.

## Funding

This work was funded by the 135 Project of Outstanding Development of West China Hospital, Sichuan University (Grant number ZY2017307).

## Conflict of interest

The authors declare that the research was conducted in the absence of any commercial or financial relationships that could be construed as a potential conflict of interest.

## Publisher’s note

All claims expressed in this article are solely those of the authors and do not necessarily represent those of their affiliated organizations, or those of the publisher, the editors and the reviewers. Any product that may be evaluated in this article, or claim that may be made by its manufacturer, is not guaranteed or endorsed by the publisher.
